# The Relationship Between Body Composition, Anaerobic Performance and Sprint Ability of Amputee Soccer Players

**DOI:** 10.2478/v10078-012-0088-3

**Published:** 2012-12-30

**Authors:** Ali Özkan, Gürhan Kayıhan, Yusuf Köklü, Nevin Ergun, Mitat Koz, Gülfem Ersöz, Alexandre Dellal

**Affiliations:** 1 School of Physical Education and Sports, Bartın University, Bartın, Turkey.; 2 School of Physical Education and Sports, Sakarya University, Sakarya, Turkey.; 3 School of Sports Science and Technology, Pamukkale University, Denizli, Turkey.; 4 Department of Physical Therapy and Rehabilitation, Hacettepe University, Ankara, Turkey.; 5 School of Physical Education and Sports, Ankara University, Ankara, Turkey.; 6 Santy Orthopedicae clinical, Medical Centre Excellence FIFA, sport science and research department, Lyon, France.; 7 Scientific Research Unit, National Centre of Medicine and Science in Sports, Tunis, Tunisia.

**Keywords:** body composition, anaerobic performance, sprint, amputee athletes

## Abstract

The purpose of the present study was to investigate the relationship between body composition, anaerobic performance and sprint performance of amputee soccer players. Fifteen amputee soccer players participated in this study voluntarily. Subjects’ height, body weight, body mass index, body fat percentage (Jackson and Pollock formula) and somatotype characteristics (Heath-Carter system) were determined. The sprint performance at 10m, 20m and 30m was evaluated, whereas the counter movement jump (CMJ), relative CMJ (RCMJ), squat jump (SJ) and relative SJ (RSJ) tests were used for the determination of anaerobic performance. The results of the Pearson Product Moment correlation analysis indicated that body composition was significantly correlated with CMJ and SJ (p < 0.01), on the other hand, no measure of body composition was significantly related to the other component (p > 0.05). A significant correlation was found between CMJ, RCMJ, SJ, 10 m, 20 m and 30 m sprint performance (p < 0.05); whereas, in contrast, no measure of body composition was significantly related to the 10 m, 20 m and 30 m sprint performance (p > 0.05). In conclusion, the findings of the present study indicated that sprint performance was described as an essential factor in anaerobic performance whereas body composition and somatotype play a determinant role in anaerobic and sprint performance in amputee soccer players.

## Introduction

In amputee soccer, short bursts of high intensity power production play a major role in performance. Amputee soccer activities are comprised of varying explosive movements like forward and backward shuffles, runs at different intensities and sustained forceful contractions to control the ball against defensive pressure. Differences in age, stature, body mass and body mass index have been recently identified between elite players of different playing positions suggesting that the physical and technical demand in match-play varied for various positions (Bloomfield et al., 2007; Gomes et al., 2006). It can be suggested therefore, that anaerobic performance and the ability to perform high-intensity actions are crucial in this type of sport (Iaia et al., 2009; Dellal et al., 2011). Anaerobic performance is composed of anaerobic power and capacity. Anaerobic power reflects the ability to use the phosphagenic system and anaerobic capacity reflects the ability to derive energy from a combination of anaerobic glycolysis and the phosphagen system. Anaerobic performance depends on many factors, such as body composition, age, sex, muscle fiber composition, muscle cross sectional area, strength and training (Kin-İşler et al., 2008).

Body composition (body size and somatotype) is another factor that is generally accepted to have a great influence on athletic performance (Reilly et al., 2000; Gomes et al., 2005). Specifically body fat and fat free mass have been accepted as crucial components of anaerobic performance (Mayhew et al., 2001) and sprint performance (Dowson et al., 1998; Young et al., 1995). For instance, Mayhew et al. (2001) reported that body composition component was one of the major factors explaining anaerobic power and sprint performance (Jacobs et al., 1987).

Sprint performance is another fundamental activity for many sports and consists of a number of components such as the start, acceleration and maximum speed phases. Sprinting also requires high force production (Mero et al., 1992). Previous research has identified force production capabilities of legs to be a key component in sprinting (Kin-İşler et al., 2008). However, these studies used only single-trial sprint protocols, neglecting to address the repeated-effort sprint requirements specific to the nature of many field and court sports. The relationship between the force-generating capacity of muscles and repeated-sprint ability has received little attention (Kin-İşler et al., 2008).

Amputee soccer is gaining popularity throughout the world and it represents a game that places demand on anaerobic performance, muscular strength, sprint performance, balance and locomotor capacity. In amputee soccer, matches are played between teams of seven players using bilateral crutches. Wearing a prosthetic device is not allowed during match play (Yazıcıoglu et al., 2007a). The match is played in two equal periods of 25 minutes each. Play may be suspended for “time-outs” of one per team per half which must not exceed one minute. The half time interval must not exceed 10 minutes (Yazıcıoglu et al., 2007b). These rules emphasize the importance of body composition, anaerobic performance and speed of action, three different variables that have not been hitherto studied within this frame. Therefore, the purpose of the present study was to investigate the relationship composition, anaerobic performance and sprint performance of amputee soccer players.

## Methods

### Subjects

Fifteen male amputee soccer players with unilateral below-knee amputation participated in this study voluntarily. The causes of amputation were gun shot in 13 subjects, traffic accident in one subject and congenital malformation in one subject. Their mean age, height, body mass and body fat were 25.5 ±5.8 yrs, 169.8 ± 5.5 cm, 66.5 ± 10.2 kg and 10.1 ± 3.6 %, respectively. The study group consisted of active football players of the amputee football team and all the players were the members of the same team competing in Amputee Super League and trained for two hours five days per week. Subjects’ mean training experience was 3.3 ± 2.9 yrs. Subjects were informed about the possible risks and benefits of the study and gave informed consent to participate in this study.

### Procedures

#### Anthropometric Measurements

The body height of the soccer players was measured by a stadiometer with an accuracy of ± 1 cm (SECA, Germany), and an electronic scale (SECA, Germany) with an accuracy of ± 0.1 kg was used to measure body mass. Skinfold thickness was measured with a Holtain skinfold caliper (Hotain, UK) which applied a pressure of 10 g/mm^2^ with an accuracy of ± 2 mm. Gulick anthropometric tape (Holtain, UK) with an accuracy of ± 1 mm was used to measure the circumference of extremities. Diametric measurements were determined by Harpenden calipers (Holtain, UK) with an accuracy of ± 1 mm. The soccer players’ somatotypes were then calculated using the Heath-Carter formula (1990) and the percentage of body fat was determined by the Jackson and Pollock formula (1978).

#### Anaerobic performance evaluation (Vertical jump tests)

All jumps were performed using a force plate (Sport Expert TM, MPS-501 multi purpose measurement system, Tumer Electronic LDT, Turkey). After a familiarization session (learning the proper techniques of the two jump conditions), each subject performed 3 maximal CMJs and SJs, with approximately 2 minutes recovery in between. The subjects did not use bilateral crutches (wearing a prosthetic device was not allowed during jumping). Players were asked to jump as high as possible; the best score was recorded in centimeters. The SJ was performed from a starting position with the subjects’ knees flexed to 90°, hands fixed on the hips and with no allowance for preparatory counter movement. The CMJ was performed from an upright standing position, with the hands fixed on the hips and with a counter movement preparatory phase which ended at a position corresponding to the starting position in the SJ. For the SJ and CMJ, two parameters were estimated: maximum jumping height and total work produced by the body in each jumping condition calculated according to the Genuario and Dolgener formula (1980).

#### Sprint performance evaluation

The sprint performance of the amputee soccer players was evaluated using three tests: a 10, 20 and 30 m single-sprint test. Sprint times were measured with light gates combined to the timing system (Prosport, Tumer Electronics, Ankara, Turkey). For the 4 single sprint tests, the timing light gates were placed at the start and at the finish (10, 20 and 30 m mark). These tests were performed in an indoor court to eliminate environmental conditions. The subjects performed two maximal trial sprints using bilateral crutches (wearing a prosthetic device was not allowed during the sprint) over the 10, 20 and 30 m distances with one minute rest intervals. The best time performance at each distance was used for further evaluations.

### Statistical Analyses

The data are reported as means and standard deviations. Before using parametric tests, the assumption of normality was verified using the Shapiro-Wilk test. Then, the relationships between body composition, anaerobic performance and sprint performance were evaluated by the Pearson Product Moment Correlation analysis. All analyses were executed in SPSS for Windows version 10.0 and the statistical significance was set at p < 0.05.

## Results

Body composition, anaerobic performance and sprint performance of amputee soccer players are displayed in Tables 1 and 2, respectively.

Correlations between body composition, anaerobic and sprint performance are presented in Table 3. As seen in Table 3, body composition was significantly correlated with CMJ and SJ, on the other hand, no measure of body composition was significantly related to the other component (p > 0.05).

According to the Pearson Product Moment correlation analysis, significant correlation was found between CMJ, RCMJ, SJ, 10, 20 and 30 m sprint performance; whereas, in contrast, no measure of body composition was significantly related to the 10, 20 and 30 m sprint performance (p > 0.05).

## Discussion

The main findings of the present study suggest that there is a moderate correlation between fat %, CMJ, SJ and sprint performance. It is already known that muscular strength is one of the important factors that has a major role in anaerobic and sprint performance. This is because with increased muscular strength the ability of muscles to generate power in short-term high intensity activities (10, 20, 30 m sprint) also increases. This result is consistent with the results of previous studies. A number of studies examined the relationship between walking-sprint ability and muscular strength (Klingenstierna et al., 1990; Moirenfeld et al., 2000). From the study of Nadollek et al. (2002), it became clear that strong hip muscles were correlated with increased weight-bearing on the amputated limb and improved gait-sprint parameters (cadance, gait cycle, velocity, step length and stride length). Velzen et al. (2006) investigated the relationship between physical capacity (aerobic capacity, anaerobic capacity, muscle force, flexibility and balance) and walking ability in lower limb amputation and determined a strong positive correlation between walking ability and muscular strength. In addition, a relationship between muscular strength and vertical jump performance (CMJ and SJ) was found by different authors (Çakır et al., 2009; Paasuke et al., 2001; Tsiokanos et al., 2002). Furthermore, Thorland et al. (1987) found a significantly strong correlation between isokinetic knee strength and anaerobic power and capacity of female sprinters and middle distance runners. This implies that strength plays a major role in high intensity activities (especially sprinting and jumping).

When performing different types of jumps, the central nervous system uses different motor programs to execute the neuromuscular coordination necessary for the specific jumps. The SJ can be used as the most basic functional expression of explosive muscle strength as it requires only concentric activation. The CMJ requires moderate eccentric activation followed by high concentric activation, and therefore requires a more complex timing and graded recruitment of motor units. Thus, the SJ can serve as a baseline for the potential of explosive muscle strength and CMJ may indicate the development of this potential (Benckeet al., 2002). Most of the studies mentioned above demonstrated a significant correlation between different sprint performance and both SJ and CMJ performance (Kin-İşler et al., 2008). We were able to corroborate a relationship between sprint performance and vertical jump performance (CMJ-SJ). It has been suggested that when the subjects’ body mass is incorporated, this leads to a more representative indicator of the subjects’ jumping abilities. This means that for the same jumping height a heavier subject will need greater sprint performance to overcome the higher external resistance during jumping. Another finding of the present study was that the measure of the jump performance was significantly negatively related to the single-sprint performance. Similarly, Jason and Mc Guıgan (2008) determined an association between the CMJ and single-sprint performance through the 9.1 m, 18.2 m, pro-agility and Illinois tests, and it is known that the CMJ and SJ are typically used as indicators of lower body power. This jump performance was inversely related to sprint times with the relationship becoming increasingly stronger with increasing sprint distances (Jason and Mc Guıgan, 2008).

Conversely, Cronin and Hansen (2005) found that CMJ and SJ measures were significantly correlated to sprint performance and Hennessey and Kilty (2001) also demonstrated that CMJ and SJ were related to all three distances.

Amputee soccer is a developing sport branch in Turkey and is played at the league level. Hence, the subjects of the present study are amateur players with training experience of 2.6 ± 1.7 yrs in amputee soccer. Having low training experience may be one of the reasons for not finding an association between body composition, anaerobic performance and single-sprint performance. One possible explanation for the lack of association may be the different energy systems that each measure demands. The findings of the present study indicated that body composition and somatotype play a significant role in anaerobic and sprint performance. In addition, sprint performance was found to be an important factor in anaerobic performance of amputee soccer players.

## Figures and Tables

**Table 1:** Body composition and somatotype characteristics of amputee soccer players (mean ± sd)

Amputee soccer players (n=15)	Body Height (cm)	Body Mass (kg)	Body Fat (%)	Endomorfism	Mesomorfism	Ectomorfism
	169.8±5.5	66.5±10.2	10.1±3.6	3.11±0.98	4.74±1.26	2.45±1.93

**Table 2:** Anaerobic performance and sprint performance values of amputee soccer players (mean ± sd)

	Counter Movement Jump			Squat Jump			Sprint Performance		

Amputee soccer players (n=15)	Absolute (CMJ) (Watt)	Relative (RCMJ) (W·kg^−1^)	Jump Height (CJH) (cm)	Absolute (SJ) (Watt)	Relative (RSJ) (W·kg^−1^)	Jump Height (SJH) (cm)	10m (s)	20m (s)	30m (s)
	837.6±198.9	12.5±1.8	33.0±9.7	809.2±177.9	12.2±1.9	31.2±10.1	2.06±0.2	3.7±0.4	5.4±0.7

**Table 3:** Correlations between body composition, anaerobic and sprint performance of amputee soccer players

	CMJ (Watt)	RCMJ (W·kg^−1^)	SJ (Watt)	10 (m)	20 (m)	30 (m)
Fat %	0.756^**^	NS	0.674^**^	NS	NS	NS
Endomorfism	0.696^**^	NS	0.659^**^	NS	NS	NS
Mesomorfism	NS	NS	NS	NS	NS	NS
Ectomorfism	^−^0.661^**^	NS	NS	0.613^*^	NS	0.649^**^
10m	^−^0.683^**^	^−^0.552^*^	^−^0.556^**^	NS	NS	NS
20m	^−^0.585^**^	^−^0.593^*^	^−^0.581^*^	NS	NS	NS
30m	^−^0.661^**^	^−^0.604^*^	NS	NS	NS	NS

* p<0.05;

** p<0.01;

NS: No Significant
